# Control of intervalley scattering in Bi_2_Te_3_ via temperature-dependent band renormalization

**DOI:** 10.1038/s41535-025-00842-8

**Published:** 2026-01-09

**Authors:** A. Jabed, F. Goto, B. Frimpong, D. Armanno, A. Longa, M. Michiardi, A. Damascelli, P. Hofmann, G. Jargot, H. Ibrahim, F. Légaré, N. Gauthier, S. Beaulieu, F. Boschini

**Affiliations:** 1 Advanced Laser Light Source, Institut National de la Recherche Scientifique, Varennes, QC Canada; 2 Université de Bordeaux-CNRS-CEA, CELIA, UMR5107, Talence, France; 3 Quantum Matter Institute, University of British Columbia, Vancouver, BC Canada; 4 Department of Physics & Astronomy, University of British Columbia, Vancouver, BC Canada; 5 Department of Physics and Astronomy, Interdisciplinary Nanoscience Center, Aarhus University, Aarhus C, Denmark

**Keywords:** Materials science, Physics

## Abstract

The control of out-of-equilibrium electron dynamics in topological insulators is essential to unlock their potential in next-generation quantum technologies. However, the role of temperature on the renormalization of the electronic band structure and, consequently, on out-of-equilibrium electron scattering processes is still elusive. Here, using high-resolution time- and angle-resolved photoemission spectroscopy (TR-ARPES), we show that even a modest (~15 meV) renormalization of the conduction band of Bi_2_Te_3_ can critically affect bulk and surface electron scattering processes. Supported by kinetic Monte Carlo simulations, we show that temperature-induced changes in the bulk band structure modulate the intervalley electron-phonon scattering rate, reshaping the out-of-equilibrium response and the long-lasting charge accumulation at the bottom of the conduction band. This work establishes temperature as an effective control knob for engineering scattering pathways in topological insulators.

## Introduction

Three-dimensional topological insulators (TIs), owing to the presence of a topologically-protected metallic surface state (TSS) within an insulating bulk energy gap^1–6^, are still prime candidates for advanced technological applications, such as photogalvanic current generation^7^, (opto-)spintronics^8–10^, and quantum computing^11–13^. In this regard, the common denominator of all TI-based devices is the demand for precise control of their electronic properties and response to external stimuli^14–16^, in both perturbative and non-perturbative regimes. This desire is contingent upon the need for a deeper understanding of their out-of-equilibrium charge dynamics.

To this end, time- and angle-resolved photoemission spectroscopy (TR-ARPES) is nowadays a well-established tool for probing ultrafast electron dynamics in quantum materials with remarkable temporal, energy and momentum resolutions^17,18^. When applied to TIs, TR-ARPES has provided direct evidence for electron relaxation processes in the TSS well beyond the timescale of a few ps^19–23^, results that have been discussed in terms of the intrinsically weak electron-phonon coupling caused by the limited scattering phase space in concert with the topological protection of the TSS against large-momentum scattering^24–28^. However, although it is widely accepted that bulk bands act as charge reservoirs for the TSS^29–32^, it is still unclear how their specific electronic dispersion may impact ultrafast electron relaxation dynamics. In this context, Chen et al.^33^ have shown how ultrafast scattering processes in p-doped Bi_2_Te_3_ depend closely on the pump photon energy, i.e., on the specific unoccupied states that are populated by photoexcited electrons. In particular, they reported that photoexcitation with 330 meV photon energy results in slow carrier dynamics dominated by intervalley scattering.

Here, we present a high-resolution TR-ARPES study of p-doped Bi_2_Te_3_ and, with the support of kinetic Monte Carlo (KMC) simulations, we offer first evidence of how a minor renormalization of the bulk dispersion has a substantial impact on the ultrafast electron dynamics. Indeed, as the sample temperature increases, the interplay between thermal expansion and electron-phonon coupling results in a renormalization of the conduction band (CB)^34^, opening a new pathway for efficient intervalley scattering towards the center of the Brillouin zone (Γ). Our experimental evidence is reminiscent of what is found in semiconducting transition metal dichalcogenides, where the charge dynamics is dominated by intra- and inter-valley scattering processes and the relative energy position of the different valleys influences population lifetime^35–37^. Although a temperature-induced reduction of the energy gap in Γ has already been reported^38,39^, our results underscore the crucial, and fairly unexplored, combined role of temperature and the bulk band structure in feeding carriers to the technologically relevant TSS. Furthermore, as the temperature increases, the reduction of the direct bulk gap, i.e., at the Γ point, comes hand-in-hand with an enhancement of the intervalley electron-phonon coupling. Since the Seebeck coefficient depends strongly on the electronic distribution around the Fermi level, the here-reported temperature dependence of the bulk gap (and its impact on charge accumulation) may be key to the understanding of the remarkable thermoelectric performance of Bi_2_Te_3_^34,40–43^.

## Results

### Time-resolved ARPES mapping of the unoccupied states of p-doped Bi_2_Te_3_

**Fig. 1:**
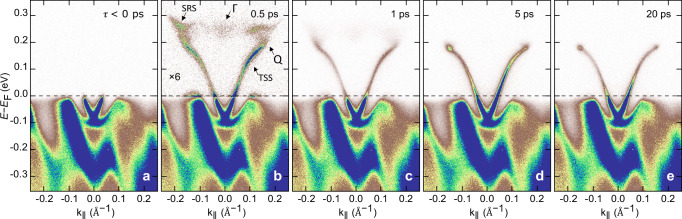
Long-lasting intensity buildup in the unoccupied states of p-doped Bi_2_Te_3_. Spectra acquired (6 eV probe/300 meV pump) at a temperature of 30 K, along the Γ-M direction, for different pump-probe delays: **a** *τ* < 0 ps, **b** 0.5 ps, **c** 1 ps, **d** 5 ps and **e** 20 ps. The intensity above the Fermi level in (**b**) has been multiplied by a factor of 6 to enhance visibility. See Supplementary Information for details of the experimental geometry.

Figure 1 displays TR-ARPES maps acquired at 30 K using 6 eV probe and 300 meV pump pulses (details in “Methods”), along the Γ-M direction, for different pump-probe delays (additional data at different temperatures are provided in Fig. S2). In agreement with ref. ^33^, the 300 meV pump excitation enables direct promotion of electrons from the valence band (VB) into the CB of Bi_2_Te_3_. In particular, we report a direct optical transition into the surface resonance state^44–46^, slightly off the Γ point.

By simple visual inspection of the ARPES map at 0.5 ps pump-probe delay (Fig. 1b), it is clear that the pump pulse populates a continuum of states connecting the CB absolute minimum at ± 0.15 Å^−1^(Q-valleys) and the CB local minimum at the Γ point (Γ-valley). While the CB signal at Γ decays within a few ps, an intensity buildup forms and persists for tens of ps at the bottom of the Q-valleys, where the TSS merges with the CB. This intensity buildup has been previously reported and attributed to the first evidence of a spatially indirect topological exciton that forms by binding surface electrons (sitting on the TSS) with bulk holes (at the VB maximum)^47^.

We verified that this intensity buildup is also observed with 6.2 eV-probe/1.55 eV-pump photon energies, as well as that it fades with increasing temperature (see Supplementary Information, Figs. S2 and S4), as would also be expected in the exciton formation scenario^47^. However, as detailed below, we show that the observed temperature dependence is also consistent with the interpretation of these spectral features as bulk band states, and it can be directly linked to the efficiency of the intervalley scattering between bulk state valleys. We also note that when the TSS is in close proximity to the bulk states, it could possibly inherit their orbital character^47^. In fact, (i) the intensity buildup displays a trigonal pattern reminiscent of the bulk states (*k*_*x*_-*k*_*y*_ iso-energy contour maps in Fig. S1), and (ii) the linear dichroism maps (inspired by refs. ^48–50^, see Fig. S1) hint at different orbital characters between the intensity buildup and the surrounding TSS. Based on the discussion above, and since our conclusions do not depend on the existence of the spatially indirect exciton, we adopt the simplest possible model. We then refer to the intensity buildup as a direct signature of the carrier accumulation at the bottom of the Q-valley, and we establish its population density as a measure of the strength of the Γ ↔ Q intervalley electron-phonon scattering channel.

### Temperature dependence of bulk band dispersion and ultrafast electron dynamics

**Fig. 2:**
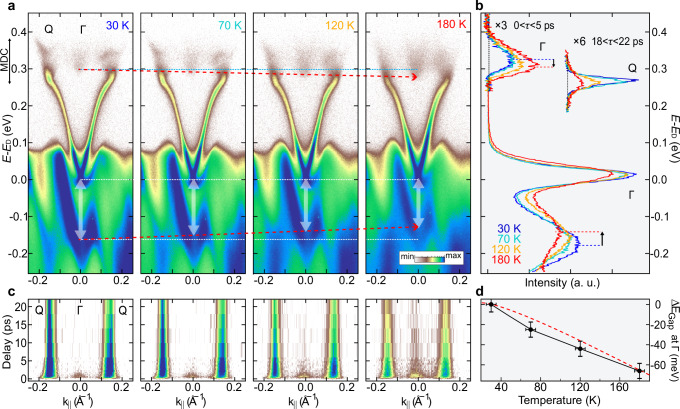
Temperature-induced bulk band renormalization. **a** Temperature-dependent TR-ARPES results on p-doped Bi_2_Te_3_, along the Γ-M direction, and integrated over the first 5 ps. **b** Energy distribution curves at the Γ point (*k*_∥_ ~ 0 ±50 mÅ^−1^, up to 5 ps), and at the Q-valley (*k*_∥_ ~ − 150 ± 25 mÅ^−1^, at 20 ps). **c** Momentum distribution curves (MDCs) as a function of pump-probe delay at the corresponding temperatures. The integration window for MDCs is indicated by the double-headed black arrows in (**a**). **d** Relative change of the direct bulk band gap at Γ as a function of the temperature. The red dashed line displays ab-initio predictions, digitized from ref. ^34^.

**Fig. 3:**
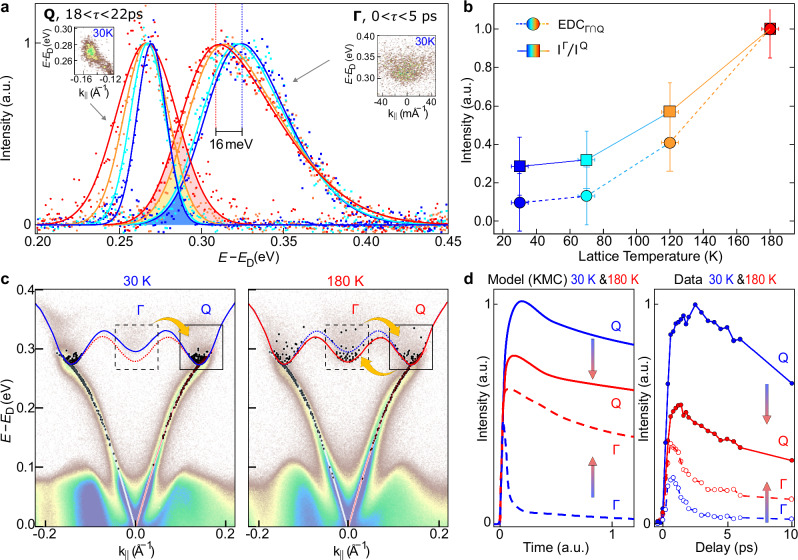
Intervalley scattering is behind the emergence of the intensity buildup. **a** Normalized Energy Distribution curves at different temperatures with superimposed an asymmetric gaussian fit for the Γ (0 < *τ* < 5 ps) and Q valleys (18 ps < *τ* < 22 ps). The spectral overlap is indicated as shadow areas with different colors for each temperature. **b** The spectral overlap between the Γ- and Q-valley, ${EDC}_{\Gamma \cap Q}={I}_{norm}^{Q}\times {I}_{norm}^{\Gamma }$ (colored circles), is compared with the ratio of the photoemission intensities for the two valleys (colored squares). **c** Electron occupancy obtained by the electron-phonon scattering simulations at 30 K and 180 K, for the last delay, superimposed on top of the experimental band structure from Fig. 2. **d** Comparison between simulated and experimental electron dynamics at Γ (dashed lines) and Q (solid lines) valleys. The band structure renormalization increases the efficiency of intervalley scattering from Q to Γ.

We now move on exploring the role of the temperature as tuning knob for the intervalley scattering in the CB. To do so, Fig. 2a displays ARPES maps integrated over the first 5 ps pump-probe delay at different temperatures (note that for comparison purposes, the energy axis is scaled with respect to the Dirac point position, E_D_, as in refs. ^39,47^). At the Γ point, where the temperature-induced renormalization of the band structure is more pronounced, we report an upward (downward) shift in the energy of the VB (CB) maximum (minimum), as the temperature increases. Interestingly, according to ref. ^34^, the gap at the Γ-point is extremely sensitive, in band-inverted materials like TIs, to both thermal expansion and electron-phonon coupling. Therefore, we inspect the energy distribution curves (EDCs) at different temperatures (Fig. 2b), from which we extract the variation of the energy gap (Fig. 2d). As the temperature increases from 30 K to 180 K, the band gap at Γ reduces by ~ 62 meV, in good agreement with the ab-initio theoretical estimate of ref. ^34^, where thermal expansion and electron-phonon coupling contributions have been accounted for (see red dashed line in Fig. 2d). In contrast, the peak position of the EDCs at the Q-valley, where the TSS is affected by trigonal-warping and merges into the CB^1^, remains essentially unchanged across all temperatures (~ 3 meV shift), as shown in Fig. 2b and Fig. 3a.

It is now instructive to track how the electronic population evolves at Γ and Q as a function of temperature. To do so, in Fig. 2c we show the transient evolution of the momentum distribution curves for four temperatures, in an energy range that includes the entire CB population (vertical line on the left of panel a). At low temperature, the Γ-valley spectral weight vanishes within a few ps and the Q-valley dominates the dynamics. As the temperature increases, a long-lasting slowly-decaying signal appears at the Γ-valley and the spectral weight at the Q-valleys is reduced, in good agreement with what is observed in the EDCs of Fig. 2b (see also Fig. 3d).

Figure 2c points towards a temperature-dependent coupling between the Γ- and Q-valleys, which is favored by changes in the CB dispersion. To be more quantitative, and identify a figure of merit that captures the role of the bulk band renormalization in defining electron dynamics, we compare normalized EDCs at the Q-valley (20 ps) and at the Γ-valley (0–5 ps delay range). In particular, we assess the spectral overlap between the Γ- and Q-valleys EDCs, illustrated by the shadow area in Fig. 3a, by evaluating the product of the two normalized EDCs (EDC_Γ∩*Q*_, colored circles in Fig. 3b). The increase in the spectral overlap corresponds to an enhancement of the scattering phase space available for an electron to escape a given valley by intervalley scattering. This is also supported by the fact that ratio of spectral intensities at Γ and Q (*I*^Γ^/*I*^*Q*^, colored squares in Fig. 3b) follows the same trend in temperature as the spectral overlap EDC_Γ∩*Q*_, thus indicating a transfer of spectral weight from the Q- to the Γ-valley mediated by intervalley electron-phonon scattering.

### Temperature-induced changes of the scattering phase space

To better interpret our experimental evidence and the interplay between the Γ- and the Q-valleys, we implemented a simulation for electron-phonon scattering using a KMC approach, similar to ref. ^51^, but with momentum resolution^52^. We run KMC calculations for two different electronic band structures, namely at 30 K and 180 K (red and blue curves in Fig. 3c), that only differ by a small shift of ~16 meV of the CB minimum at Γ, based on the experimental observation (see Fig. 2a). We model the CB using a discrete distribution of equally spaced electronic states, and we tune the mesh density of the simulations such that the spectral overlap between the KMC occupancies, Γ ∩ *Q*, approximately follows the temperature dependence of the spectral overlap extracted by TR-ARPES data (circles in Fig. 3b). In the KMC calculations, we impose a Gaussian distribution of N electrons centered at 0.4 eV above the Dirac point (to match the experimental condition at *τ* ~ 0.5 ps, see Figs. 1, 2), and then evaluate the electron occupancy as a function of time, *f*_*i*_(*t*), by solving the rate equation:

1 $$\frac{\partial {f}_{i}}{\partial t}=\mathop{\sum }\limits_{j}[{W}_{j,i}{f}_{j}(1-{f}_{i})-{W}_{i,j}{f}_{i}(1-{f}_{j})],$$

where *W*_*i*,*j*_ is the probability for each electron (*k*_*i*_, *E*_*i*_) to have a transition into an unoccupied state (*k*_*j*_, *E*_*j*_) by absorption or emission of a phonon, provided by the Fermi’s golden rule (see “Methods”). Black circles in Fig. 3c display the electronic distributions obtained via KMC for 30 K and 180 K when a quasi-equilibrium condition is reached (see also Fig. S3 for different snapshots of the simulation). Figure 3d offers also direct comparison between experimental and KMC time traces at Γ- and Q-valleys (integration boxes shown in Fig. 3c). Not only do the simulated time traces successfully reproduce the transfer of spectral weight from the Q to the Γ valley at higher temperatures, they also capture the delayed occupation of the Q valley compared to the Γ valley. This delayed occupation of the Q valley, particularly at low temperatures, is directly linked to inefficient scattering from the Γ valley: the smaller the electron-phonon scattering phase space, the slower the intensity build-up at the Q valley (see Supplementary Information for further details).

Overall, the simulation successfully captures (i) the formation of the characteristic long-standing intensity buildup at the Q-valley at low-temperature (Fig. 3c), and (ii) the transfer of spectral weight from the Q- to Γ-valley at high temperature (see Fig. 3d), due to the increase of the electron-phonon scattering phase space caused by the thermal renormalization of the electronic band structure.

## Discussion

This TR-ARPES work demonstrates that temperature-induced renormalization of the CB in Bi_2_Te_3_ plays a critical role in dictating ultrafast electron dynamics. Indeed, as temperature rises, the downward shift of the CB minimum at Γ enhances the spectral overlap between the Q- and Γ-valleys. This modification opens a novel scattering channel that facilitates intervalley electron-phonon scattering, as evidenced by the marked transfer of spectral weight from Q to Γ valleys. The experimental trends are supported by KMC simulations, which well reproduce the evolution of the photoemission intensity. Our findings suggest that the long-lasting intensity buildup does not necessarily involve the emergence of a spatially indirect exciton, but its formation is well captured by the temperature-induced change in the intervalley scattering efficiency within the CB. Ultimately, our work provides a comprehensive understanding of the intricate mechanisms governing the out-of-equilibrium response of TIs, revealing how temperature-induced changes in the bulk band structure can dramatically alter their surface and bulk carrier dynamics. This approach unravels the interplay between carrier density, phonon population, and band structure renormalization, and it can also be applied to other bulk semiconducting compounds with a multi-valley landscape, including two-dimensional layered semiconductors and other three-dimensional TIs, which exhibit unique thermoelectric properties. These studies may provide valuable insights that could inform the rational design of advanced thermoelectric and quantum devices, in which precise control of out-of-equilibrium electron dynamics across a broad temperature range is critical.

## Methods

### Samples

The Bi_2_Te_3_ crystals were grown from the elements in quartz ampoules using the Bridgman method, as described elsewhere^25^.

### TR-ARPES

TR-ARPES experiments were performed at the TR-ARPES endstation of the Advanced Laser Light Source (ALLS) laboratory^53^. The sample was photoexcited with 100 fs, p-polarized, mid-IR pulses (300 meV photon energy), and the photoemission process was elicited by 6 eV, s-polarized, pulses. Photoelectrons were detected with an ASTRAIOS 190 hemispherical analyzer, and the overall energy and temporal resolutions of the TR-ARPES endstation were set to 15 meV and 300 fs, respectively. Throughout this work, the incident fluence of the pump pulse was set to ~30 μJ/cm^2^. Experiments were performed with a bias of −10 V, to extend the angular acceptance for photoelectrons^54^.

Complementary TR-ARPES work was also done at the UBC-Moore Centre for Ultrafast Quantum Matter, using a Scienta DA30L electron analyzer, 6.2 eV probe and 1.55 eV pump photons. The 1.55 eV fluence was ~50 μJ/cm^2^, and it was adjusted based on the different absorptions at 1.55 eV and 0.3 eV^55^. The sample temperature was 10 K (see Fig. S4).

### KMC calculations

In KMC calculations, the bulk CB was reproduced by a uniform grid of states that qualitatively captures the momentum-integrated density of states of a 3D system. Considering the absorption or emission of a phonon, the transition matrix of the system, *W*_*i*,*j*_, was obtained using the Fermi’s golden rule:

2 $${W}_{i,j}=\frac{2\pi }{\hslash }{g}_{0}^{2}\{[n({\omega }_{i,j})+1]\delta ({E}_{j}-{E}_{i}+\hslash {\omega }_{i,j})+n({\omega }_{i,j})\delta ({E}_{j}-{E}_{i}-\hslash {\omega }_{i,j})]\},$$

where $n(\omega )={({e}^{\frac{\hslash \omega }{{k}_{B}T}}-1)}^{-1}$ is the Bose-Einstein distribution, ${\omega }_{i,j}(q)\propto 1+\alpha \cdot \cos (\pi q)$ is the phonon dispersion evaluated at the scattering vector *q* = *k*_*i*_ − *k*_*j*_, and *g*_0_ is the electron-phonon matrix element (assumed constant for simplicity). Starting from a Gaussian distribution of electrons centered at 0.4 eV, we evaluate the electron occupancy as a function of time, *f*_*i*_(*t*), by solving the differential Eq. (1). Each step in the KMC simulation is weighted on the total transition rate $\Delta t=\frac{-{\mathrm{ln}}\,r}{{\sum }_{i,j}{W}_{i,j}},$ where *r* ∈ (0, 1] is a randomly sampled value.

Given the experimental evidence for intervalley scattering, we use an optical phonon dispersion centered at ~4 meV with a bandwidth of ±3 meV^56^. We also assume a constant electron-phonon matrix element for transitions within the CB and the TSS (same spin-polarized branch), and we set ${g}_{0}^{CB\to TSS}={g}_{0}/2$ to account for the spin polarization of the TSS. However, note that the particular choice of the phonon dispersion, i.e., optical or acoustic modes, and the strength of the electron-phonon matrix element, do not affect the qualitative results of the simulation as long as the (i) phonon emission dominates over phonon absorption, and (ii) phonon population differs from zero in the region of interest (0 < *q* < 0.25 Å^−1^)^51^. Within our simple KMC calculations, electron relaxation dynamics are purely driven by an increase in the electron-phonon scattering phase space. Additionally, since our pump excitation is in a perturbative regime, we neglect any ultrafast renormalization of the electron-phonon matrix element^57^.

## Supplementary information

Supplementary Information

## Data Availability

Data sets generated during the current study are available from the corresponding author on reasonable request.

## References

[1] Fu, L., Kane, C. L. & Mele, E. J. Topological insulators in three dimensions. *Phys. Rev. Lett.* **98**, 106803 (2007). 17358555 10.1103/PhysRevLett.98.106803

[2] Moore, J. E. The birth of topological insulators. *Nature* **464**, 194–198 (2010). 20220837 10.1038/nature08916

[3] Chen, Y. L. et al. Experimental realization of a three-dimensional topological insulator, Bi_2_Te_3_. *Science* **325**, 178–181 (2009). 19520912 10.1126/science.1173034

[4] Hasan, M. Z. & Kane, C. L. Colloquium: topological insulators. *Rev. Mod. Phys.* **82**, 3045–3067 (2010).

[5] Ferreira, G. J. & Loss, D. Magnetically defined qubits on 3D topological insulators. *Phys. Rev. Lett.* **111**, 106802 (2013). 25166691 10.1103/PhysRevLett.111.106802

[6] Jin, K. H., Jiang, W., Sethi, G. & Liu, F. Topological quantum devices: a review. *Nanoscale* **15**, 12787–12817 (2023). 37490310 10.1039/d3nr01288c

[7] Tao, X. et al. Pure spin current generation via photogalvanic effect with spatial inversion symmetry. *Phys. Rev. B* **102**, 081402 (2020).

[8] Haldane, F. D. M. Nobel lecture: topological quantum matter. *Rev. Mod. Phys.* **89**, 040502 (2017).

[9] Hsieh, D. et al. Observation of unconventional quantum spin textures in topological insulators. *Science* **323**, 919–922 (2009). 19213915 10.1126/science.1167733

[10] Michiardi, M. et al. Optical manipulation of Rashba-split 2-dimensional electron gas. *Nat. Commun.* **13**, 3096 (2022). 35654938 10.1038/s41467-022-30742-5 PMC9163084

[11] Qi, X.-L. & Zhang, S.-C. Topological insulators and superconductors. *Rev. Mod. Phys.* **83**, 1057–1110 (2011).

[12] Legg, H. F., Loss, D. & Klinovaja, J. Majorana bound states in topological insulators without a vortex. *Phys. Rev. B* **104**, 165405 (2021).

[13] Paudel, H. P. & Leuenberger, M. N. Three-dimensional topological insulator quantum dot for optically controlled quantum memory and quantum computing. *Phys. Rev. B* **88**, 085316 (2013).

[14] Zhao, W. X. et al. Topological phase transition in quasi-one-dimensional bismuth iodide Bi_4_I_4_. *npj Quantum Mater.* **9**, 103 (2024).

[15] Lewandowski, C., Nadj-Perge, S. & Chowdhury, D. Does filling-dependent band renormalization aid pairing in twisted bilayer graphene?. *npj Quantum Mater.* **6**, 82 (2021).

[16] Masuko, M. et al. Nonreciprocal charge transport in topological superconductor candidate Bi_2_Te_3_/PdTe_2_ heterostructure. *npj Quantum Mater.* **7**, 104 (2022).

[17] Boschini, F., Zonno, M. & Damascelli, A. Time-resolved ARPES studies of quantum materials. *Rev. Mod. Phys.* **96**, 015003 (2024).

[18] Zonno, M., Boschini, F. & Damascelli, A. Time-resolved ARPES on cuprates: tracking the low-energy electrodynamics in the time domain. *J. Electron Spectrosc. Relat. Phenom.* **251**, 147091 (2021).

[19] Crepaldi, A. et al. Evidence of reduced surface electron-phonon scattering in the conduction band of Bi_2_Se_3_ by nonequilibrium ARPES. *Phys. Rev. B* **88**, 121404 (2013).

[20] Sobota, J. A. et al. Ultrafast optical excitation of a persistent surface-state population in the topological insulator Bi_2_Se_3_. *Phys. Rev. Lett.* **108**, 117403 (2012). 22540508 10.1103/PhysRevLett.108.117403

[21] Sterzi, A. et al. Bulk diffusive relaxation mechanisms in optically excited topological insulators. *Phys. Rev. B* **95**, 115431 (2017).

[22] Neupane, M. et al. Gigantic surface lifetime of an intrinsic topological insulator. *Phys. Rev. Lett.* **115**, 116801 (2015). 26406846 10.1103/PhysRevLett.115.116801

[23] Huang, Y. et al. Ultrafast measurements of mode-specific deformation potentials of Bi_2_Te_3_ and Bi_2_Se_3_. *Phys. Rev. X* **13**, 041050 (2023).

[24] Sánchez-Barriga, J. et al. Ultrafast spin-polarization control of Dirac fermions in topological insulators. *Phys. Rev. B* **93**, 155426 (2016).

[25] Michiardi, M. et al. Bulk band structure of Bi_2_Te_3_. *Phys. Rev. B* **90**, 075105 (2014).

[26] Hajlaoui, M. et al. Tuning a Schottky barrier in a photoexcited topological insulator with transient Dirac cone electron-hole asymmetry. *Nat. Commun.* **5**, 3003 (2014). 24389793 10.1038/ncomms4003

[27] Sobota, J. A. et al. Distinguishing bulk and surface electron-phonon coupling in the topological insulator Bi_2_Se_3_ using time-resolved photoemission spectroscopy. *Phys. Rev. Lett.* **113**, 157401 (2014). 25375740 10.1103/PhysRevLett.113.157401

[28] Pan, Z.-H. et al. Measurement of an exceptionally weak electron-phonon coupling on the surface of the topological insulator Bi_2_Se_3_ using angle-resolved photoemission spectroscopy. *Phys. Rev. Lett.* **108**, 187001 (2012). 22681106 10.1103/PhysRevLett.108.187001

[29] Sánchez-Barriga, J. et al. Subpicosecond spin dynamics of excited states in the topological insulator Bi_2_Te_3_. *Phys. Rev. B* **95**, 125405 (2017).

[30] Hajlaoui, M. et al. Ultrafast surface carrier dynamics in the topological insulator Bi_2_Te_3_. *Nano Lett.* **12**, 3532–3536 (2012). 22658088 10.1021/nl301035x

[31] Papalazarou, E. et al. Unraveling the Dirac fermion dynamics of the bulk-insulating topological system Bi_2_Te_2_Se. *Phys. Rev. Mater.* **2**, 104202 (2018).

[32] Soifer, H. et al. Band-resolved imaging of photocurrent in a topological insulator. *Phys. Rev. Lett.* **122**, 167401 (2019). 31075004 10.1103/PhysRevLett.122.167401

[33] Chen, W. et al. Distinct light-matter coupling mechanisms in Bi_2_Te_3_: crossover from above-gap photoexcitation to light-field dressing. *Phys. Rev. B* **110**, L201116 (2024).

[34] Monserrat, B. & Vanderbilt, D. Temperature effects in the band structure of topological insulators. *Phys. Rev. Lett.* **117**, 226801 (2016). 27925756 10.1103/PhysRevLett.117.226801

[35] Ataei, S. S. & Sadeghi, A. Competitive screening and band gap renormalization in n-type monolayer transition metal dichalcogenides. *Phys. Rev. B* **104**, 155301 (2021).

[36] Ulstrup, S. et al. Ultrafast band structure control of a two-dimensional heterostructure. *ACS Nano* **10**, 6315–6322 (2016). 27267820 10.1021/acsnano.6b02622

[37] Lee, D. H., Choi, S. J., Kim, H., Kim, Y. S. & Jung, S. Direct probing of phonon mode specific electron–phonon scatterings in two-dimensional semiconductor transition metal dichalcogenides. *Nat. Commun.* **12**, 4520 (2021). 34312387 10.1038/s41467-021-24875-2 PMC8313722

[38] Kumar, A., Kumar, S., Miyai, Y. & Shimada, K. Temperature-dependent band modification and energy dependence of the electron-phonon interaction in the topological surface state on Bi_2_Te_3_. *Phys. Rev. B* **106**, L121104 (2022).

[39] Mori, R. et al. Possible evidence of excitonic condensation in a topological insulator. *Proc. Natl. Acad. Sci.* **122**, e2422667122 (2025). 40294261 10.1073/pnas.2422667122 PMC12067287

[40] Gibbs, Z. M., Kim, H.-S., Wang, H. & Snyder, G.J., Band gap estimation from temperature dependent seebeck measurement-deviations from the 2e relation. *Appl. Phys. Lett.* **106**, 022112 (2015).

[41] Rittweger, F., Hinsche, N. F., Zahn, P. & Mertig, I. Signature of the topological surface state in the thermoelectric properties of Bi_2_Te_3_. *Phys. Rev. B* **89**, 035439 (2014).

[42] Liang, J., Cheng, L., Zhang, J., Liu, H. & Zhang, Z. Maximizing the thermoelectric performance of topological insulator Bi_2_Te_3_ films in the few-quintuple layer regime. *Nanoscale* **8**, 8855–8862 (2016). 27071548 10.1039/c6nr00724d

[43] Cao, T. et al. Advances in bismuth-telluride-based thermoelectric devices: progress and challenges. *eScience* **3**, 100122 (2023).

[44] Hedayat, H. et al. Ultrafast evolution of bulk, surface and surface resonance states in photoexcited Bi_2_Te_3_. *Sci. Rep.* **11**, 4924 (2021). 33649414 10.1038/s41598-021-83848-z PMC7921141

[45] Cacho, C. et al. Momentum-resolved spin dynamics of bulk and surface excited states in the topological insulator Bi_2_Se_3_. *Phys. Rev. Lett.* **114**, 097401 (2015). 25793848 10.1103/PhysRevLett.114.097401

[46] Jozwiak, C. et al. Spin-polarized surface resonances accompanying topological surface state formation. *Nat. Commun.* **7**, 13143 (2016). 27739428 10.1038/ncomms13143 PMC5067600

[47] Mori, R. et al. Spin-polarized spatially indirect excitons in a topological insulator. *Nature* **614**, 249–255 (2023). 36755173 10.1038/s41586-022-05567-3

[48] Beaulieu, S. et al. Unveiling the orbital texture of 1T−TiTe_2_ using intrinsic linear dichroism in multidimensional photoemission spectroscopy. *npj Quantum Mater.* **6**, 93 (2021).

[49] Cao, Y. et al. Mapping the orbital wavefunction of the surface states in three-dimensional topological insulators. *Nat. Phys.* **9**, 499–504 (2013).

[50] Min, C. H. et al. Orbital fingerprint of topological Fermi arcs in the Weyl semimetal tap. *Phys. Rev. Lett.* **122**, 116402 (2019). 30951331 10.1103/PhysRevLett.122.116402

[51] Sobota, J. A. et al. Ultrafast electron dynamics in the topological insulator Bi_2_Se_3_ studied by time-resolved photoemission spectroscopy. *J. Electron Spectrosc. Relat. Phenom.* **195**, 249–257 (2014).

[52] Na, M. et al. Establishing nonthermal regimes in pump-probe electron relaxation dynamics. *Phys. Rev. B* **102**, 184307 (2020).

[53] Longa, A. et al. Time-resolved ARPES with probe energy of 6.0 eV and tunable MIR pump at 250 kHz. *Opt. Express* **32**, 29549 (2024). 39573142 10.1364/OE.525265

[54] Gauthier, N. et al. Expanding the momentum field of view in angle-resolved photoemission systems with hemispherical analyzers. *Rev. Sci. Instrum.* **92**, 123907 (2021). 34972440 10.1063/5.0053479

[55] Singh, D., Nandi, S., Fleger, Y., Cohen, S. Z. & Lewi, T. Deep-subwavelength resonant meta-optics enabled by ultra-high index topological insulators. *Laser Photonics Rev.* **17**, 2200841 (2023).

[56] Li, S. & Persson, C. Thermal properties and phonon dispersion of Bi_2_Te_3_ and CsBi_4_Te_6_ from first-principles calculations. *J. Appl. Math. Phys.* **3**, 1563–1570 (2015).

[57] Zheng, Y. et al. Enhanced electron-phonon coupling by delocalizing phonon states in monolayer MoS_2_. *Nano Lett.* **22**, 6102–6109 (2022).

