# Supplementary material for: Control of intervalley scattering in Bi2Te3 via temperature-dependent band renormalization
Source: NPJ Quantum Mater. 2026 Jan 9;11(1):12. doi: 10.1038/s41535-025-00842-8 (PMC12858402; doi:10.1038/s41535-025-00842-8)
Supplement: Supplementary file 1 — Supplementary Information [file 41535_2025_842_MOESM1_ESM.pdf]

Supplementary Information for: Control of intervalley scattering in Bi_2Te_3 via temperature-dependent band renormalization

A. Jabed,[1,2,*] F. Goto,[1,*] B. Frimpong,[1] D. Armanno,[1] A. Longa,[1] M. Michiardi,[3,4] A. Damascelli,[3,4] P. Hofmann,[5] G. Jargot,[1] H. Ibrahim,[1] F. Légaré,[1] N. Gauthier,[1] S. Beaulieu,[2] and F. Boschini[1,3,†]

[1]*Advanced Laser Light Source, Institut National de la Recherche Scientifique, Varennes QC J3X 1S2 Canada*

[2]*Université de Bordeaux-CNRS-CEA, CELIA,UMR5107,F33405 Talence,France*

[3]*Quantum Matter Institute, University of British Columbia, Vancouver, BC V6T 1Z4, Canada*

[4]*Department of Physics & Astronomy, University of British Columbia, Vancouver, BC V6T 1Z1, Canada*

[5]*Department of Physics and Astronomy, Interdisciplinary Nanoscience Center, Aarhus University, 8000 Aarhus C, Denmark*

[*] These authors equally contributed

[†] fabio.boschini@inrs.ca

Symmetry of the intensity buildup

Owing to the use of a hemispherical analyzer with deflector technology in concert with sample biasing, we acquired high-resolution 4D (k_x, k_y, E, τ), TR-ARPES data of p-doped Bi_2Te_3[1, 2]. Fig. S1, top, displays several iso-energy contour maps, at different central energies (±20 meV integration range) as a function of the pump-probe delay. Photoexcited electrons populate the conduction band (CB) up to 300 meV above the Fermi level (E_F), and decay and accumulates in states at ∼200 meV above E_F within 1-2 ps. These states correspond to the position in the momentum-energy space where the hexagonal warped topological surface state crosses the CB minima and the intensity buildup appears. We note a trigonal intensity pattern for the intensity buildup energy window at 0.5 ps and 1 ps pump-probe delays, reminiscent of the characteristic bulk trigonal symmetry. At 15 ps delay, the same trigonal pattern is still present up to 180 meV above E_F (±10 meV integration range

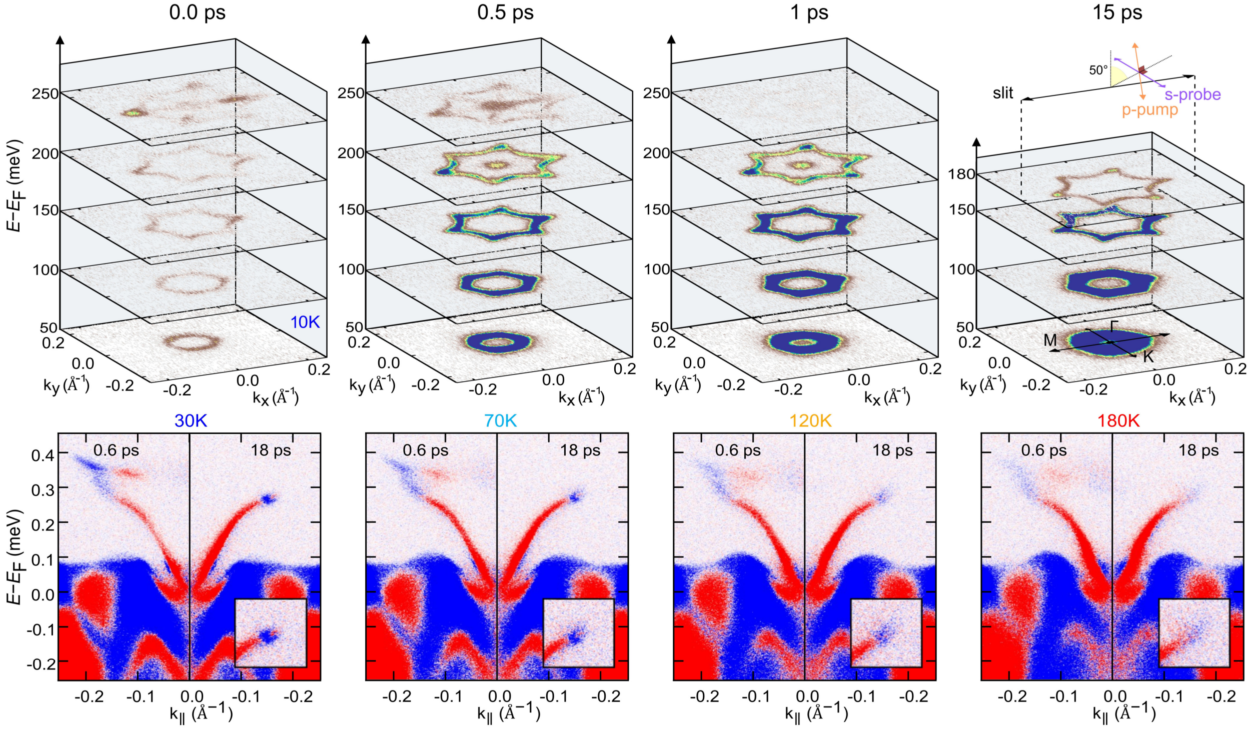

FIG. S1. **Symmetry of the intensity buildup**. Top row: 3D TR-ARPES maps in the (k_x, k_y, E) space for different pump-probe delays, from 0 to 15 ps. The experimental geometry is schematized in the inset above the 15 ps data. Bottom row: linear dichroism analysis of TR-ARPES data ($I_{LD} \propto I_{k,E} - I_{-k,E}$) for 0.6 ps and 18 ps at different temperatures.

for this central energy), indicating that the long-lasting intensity buildup holds the bulk symmetry.

Further evidence in support of the bulk nature of the intensity buildup is presented in Fig. S1, bottom. A linear dichroism analysis was performed on TR-ARPES data, with the intention of distinguishing between different orbital characters associated of the bulk and surface states. This is possible due to the precise experimental geometry used in our TR-ARPES experiments, *i.e.* s-polarized probe with mirror plane along the Γ-M direction (time-reversal invariant) [3–5]. Linear dichroism maps were generated by computing $I_{LD} \propto I_{k,E} - I_{-k,E}$. Fig. S1, bottom, displays the linear dichroism maps from 30 K to 180 K, at 0.6 ps (left) and 18 ps (right) pump-probe delays. The distinct red (positive) and blue (negative) contrast is a signature of different orbital contributions [3–5]. At 0.6 ps, the TSS exhibits a pronounced positive dichroic signal, while the CB displays a negative dichroic signal. By 18 ps, the positive dichroic contribution from the TSS intrudes into the negative dichroic region (as highlighted in the insets). This observation suggests that the TSS intersect the bulk CB at the intensity buildup location, where there is coexistence of both surface and bulk states.

Temperature dependent TR-ARPES

Fig. S2 extends the TR-ARPES results of Fig. 1 in the main text to all the temperatures investigated in this work (30 K, 70 K, 120 K, and 180 K, top to bottom) at four pump-probe delays (0.5 ps, 1 ps, 5 ps and 20 ps). The sample displayed minimal n-doping (∼10 meV) as the temperature increased (as well already discussed elsewhere [6]). To facilitate the discussion of our experimental results, the energy axis of the TR-ARPES data is referenced with respect to the Dirac point position as in [7, 8]. This same dataset is used to extract the time-integrated TR-ARPES map of Fig. 2 in the main text.

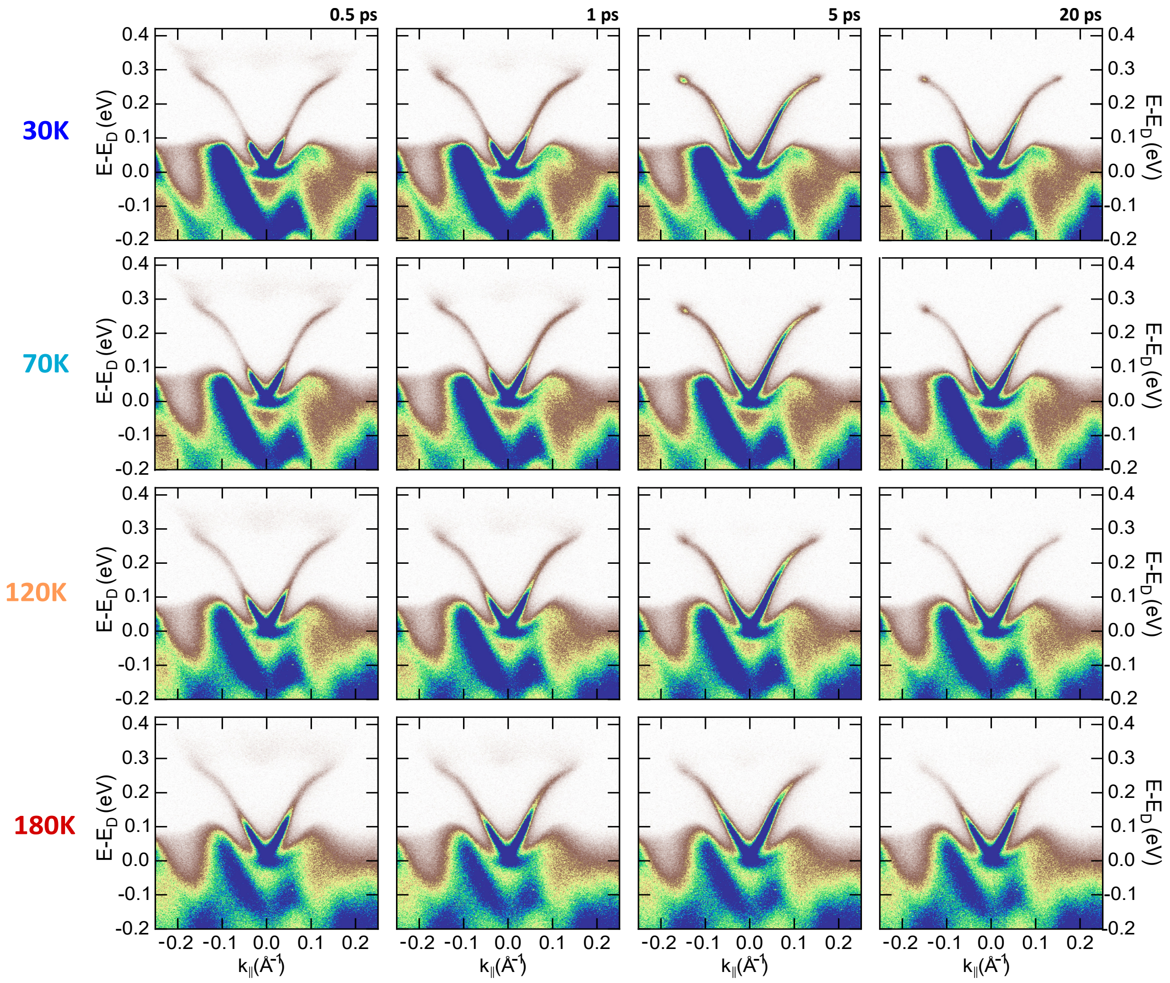

FIG. S2. **Temperature dependent TR-ARPES.** TR-ARPES spectra from 30 K to 180 K (from top to bottom) along the ΓM direction, for different pump probe delays (from 0.5 ps, on the left, to 20 ps, on the right).

Electron-phonon simulations

Fig. S3 displays different snapshots of the electron occupancy calculated using the model discussed in the main text (each black circle represents an electron in the energy-momentum space), and using two slightly different band structures of 30 K and 180 K (top and bottom row, respectively). The electron-phonon matrix element for intervalley transitions within the CB is assumed constant, g_0. Regarding electron scattering from the CB to the TSS, we set the electron-phonon matrix element to $g_0/2$ and, owing to its protection against back-scattering events, the scattering between different branches of the TSS is neglected for simplicity.

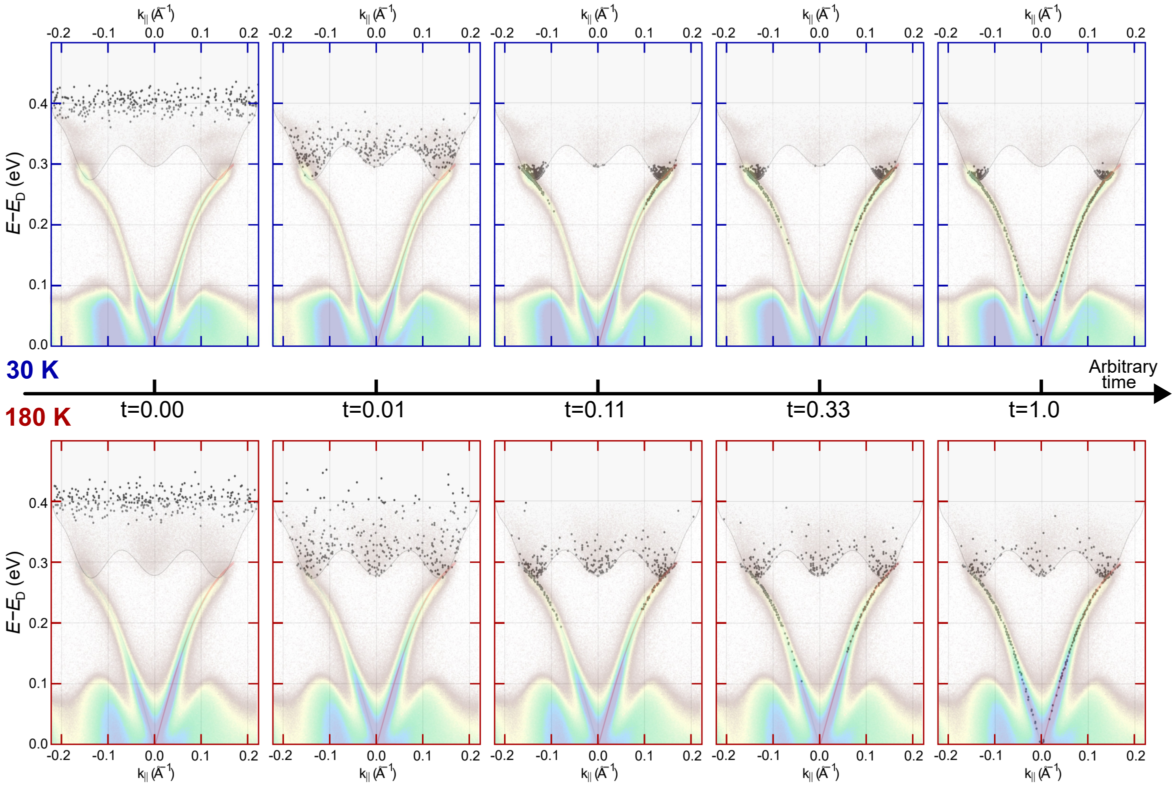

FIG. S3. **Electron-phonon simulations**. Time-resolved electron occupancies calculated by the KMC simulations at 30 K and 180 K, shown in the top and bottom row, respectively. The time axis is in arbitrary units.

As the arbitrary time of the simulation evolves, the continuous emission of phonons favors the decay of electrons in lower energy states, down to the minima of the CB where the TSS crosses the CB. Here, we report a strong bottleneck effect. Indeed, (i) the reduced

density of states of TSS compared to that of the CB, and (ii) the lack of TSS at the Γ valley, favour the formation of the characteristic intensity buildup (at high momenta) at low temperatures. However, at high temperature, the higher phonon population due to Bose-Einstein distribution, in combination with the down-shift of the CB minimum at Γ, lead to an enhancement of electron scattering towards the Γ-valley, thus reducing the occupation of the Q-valleys.

Energy position of the intensity buildup

We compare the results of two independent TR-ARPES experiments performed on p-doped Bi_2Te_3 samples from the same batch, but using different detectors (namely, SPECS ASTRAIOS 190 and Scienta DA30L), probe and pump photon energies, and base temperatures. As shown in Fig. S4a, the intensity buildup appears consistently at $272 \pm 5\,\mathrm{meV}$, as highlighted by the EDCs in Fig. S4b. We note that this value corresponds to an energy shift of more than $\sim 30\,\mathrm{meV}$ with respect to the value reported in Ref. [8], where the same spectroscopic feature is more than 0.30 eV (at 20 ps) above the Dirac point.

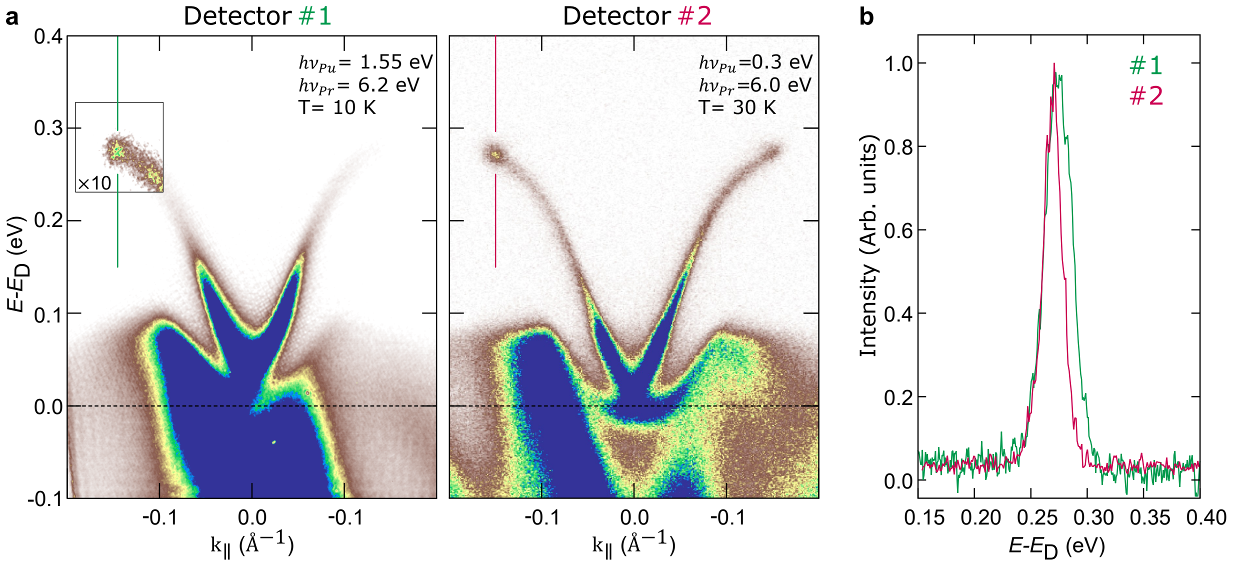

FIG. S4. **Energy position of the intensity buildup.** (a) TR-ARPES spectra at 20±2 ps acquired on Bi_2Te_3 samples using different experimental apparatus. Despite the difference in both pump-probe energies and electron detectors (Scienta DA30L on the left, and SPECS ASTRAIOS 190 on the right), the energy position of the intensity build up is comparable (within 10 meV), as indicated by the EDCs plotted on panel (b).

For a better comparison between these two TR-ARPES experiments, we estimated the number of absorbed photons within a $\Delta = 10\,\mathrm{nm}$ slab of Bi_2Te_3 for both excitation energies. Starting from the refractive index extracted from reflectivity data [9], we determined the extinction coefficient ($\alpha = 2\pi \cdot \kappa/\lambda$) and the Fresnel coefficients (R_s and R_p). The absorbed photon density per formula unit (ph/f.u.) was then calculated using

$$\eta_{f.u.} = \eta_0 \cdot (1 - R) \cdot [1 - e^{-\alpha \cdot \Delta}],$$

where $\eta_0 = F/(E_{ph} \cdot \rho \cdot \Delta)$, F is the pump fluence in μJ/cm^2, E_{ph} is the photon energy, $\rho = 5.8 \cdot 10^{21}$ f.u./cm^3 is the Bi_2Te_3 unit cell. For the s-polarized 1.55 eV pump ($F \sim 50\,\mu$J/cm^2), we obtain $\eta \approx 3.6 \cdot 10^{-3}$ ph/f.u.; for the p-polarized 0.30 eV pump ($F \sim 30\,\mu$J/cm^2), we find $\eta \approx 4 \cdot 10^{-3}$ ph/f.u.

Valence band dynamics

To highlight all contributions to the spectral weight in close proximity to the Fermi level, Fig. S5a displays the ARPES map acquired at negative delay and at a base temperature of 30 K. Due to matrix-element effects, the photoemission intensity is enhanced for the TSS (bulk) for $k_{||} > 0$ ($k_{||} < 0$), as highlighted by the linear dichroism map in panel c (see also bottom row of Fig. S1). The bottom branch of the TSS disperses in close proximity to the bulk bands up to the Fermi level (black arrows in a). Figure S5d offers a comparison between normalized MDCs at the same energy for $k_{||} > 0$ (transparent lines) and $k_{||} < 0$ (solid lines). Starting from the violet curve (0.03 eV above the Dirac point), the TSS and the bulk bands progressively merge, up to the Fermi level (black curves, 0.08 eV above the Dirac point), resulting in a spectral region with multiple spectral contributions.

Figure S5c displays EDCs at negative delay and at 3.5 ps for three momenta (green, blue, black lines in panel a).While the Dirac point does not shift (within 1 meV) or display a loss of intensity upon (green lines), the top of the VB (blue and black lines) is strongly affected by pump excitation. However, we emphasize that the interpretation of the ultrafast dynamics of the valence band maximum is not trivial since it is not possible to reliably distinguish TSS from VB contributions, as well as whether the VB is depleted or renormalized upon optical excitation (blue and black EDCs in b are practically identical upon rigid shift in energy).

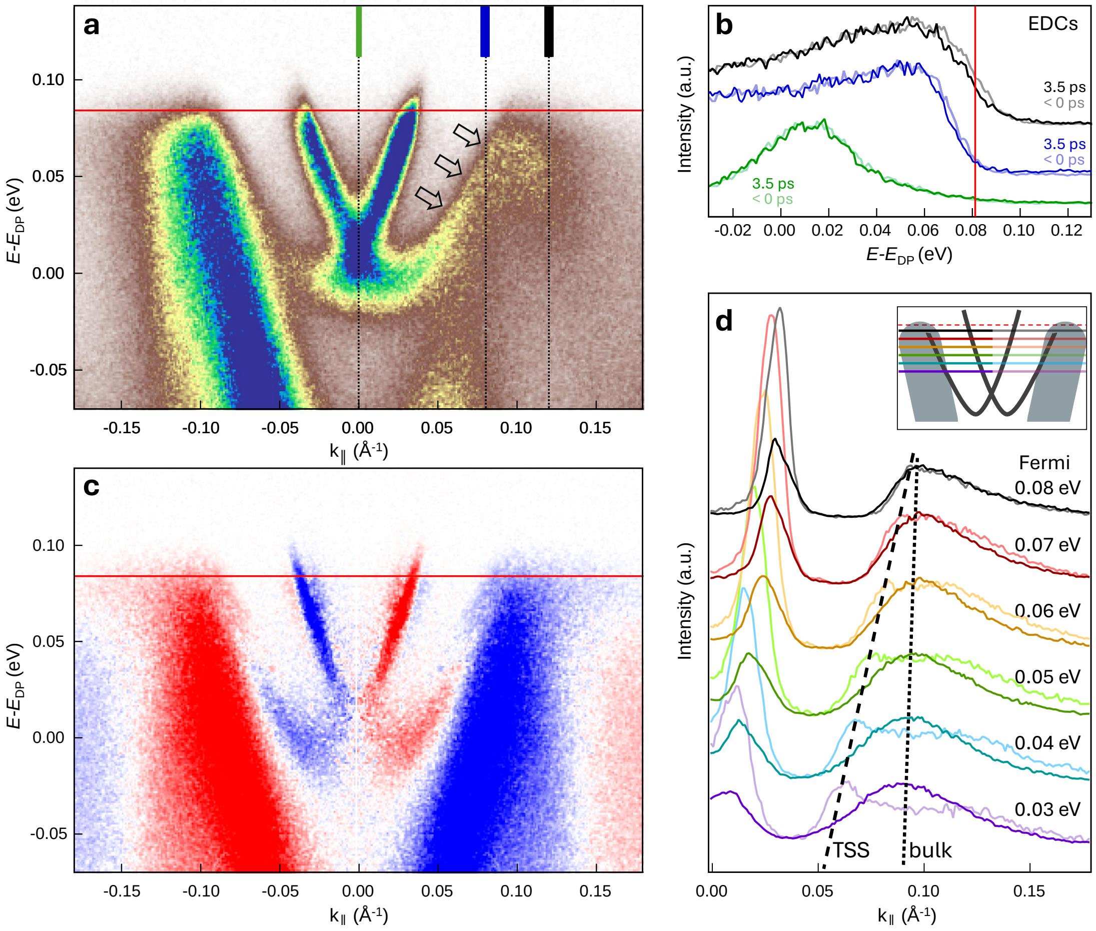

FIG. S5. **Valence band dynamics.** (a) TR-ARPES map at negative delay, 30 K. Black arrows highlight the bottom branch of the TSS. (b) EDCs along the momenta indicated in panel a. (c) The linear dichroism ARPES map (*i.e.*, $I_{k,E} - I_{-k,E}$, see Fig S2) further shows the close proximity between the bottom branch of the TSS and the VB. (d) MDCs at selected energies highlight the dispersion of the bottom branch of the TSS and the VB.

Fluence dependence of the energy gap at Γ

In order to demonstrate the crucial role of lattice temperature in determining the bulk gap at $\mathbf{\Gamma}$, Fig. S6 compares TR-ARPES spectra acquired at three pump fluences (20, 40, and 80 μJ/cm^2). For each fluence, we use the spectrum at negative delay (left panels) to evaluate the electronic temperature via Fermi–Dirac distribution fit (insets). We report only a minimal average heating effect. Right panels of the ARPES maps display the time-integrated data as in Fig. 2 of the main text (logarithmic color scale), from which we extract the energy-distribution curves at Γ (right panel). The CB and VB band-edge positions at Γ remain unchanged within the explored fluence range, despite the different photoexcited carrier densities (see inset of the right panel). These results indicate that photoexcited carriers alone do not induce the band structure renormalization; rather, the gap at Γ is controlled by lattice temperature, pointing to the key role of thermal expansion and electron–phonon coupling, in agreement with Ref. [10].

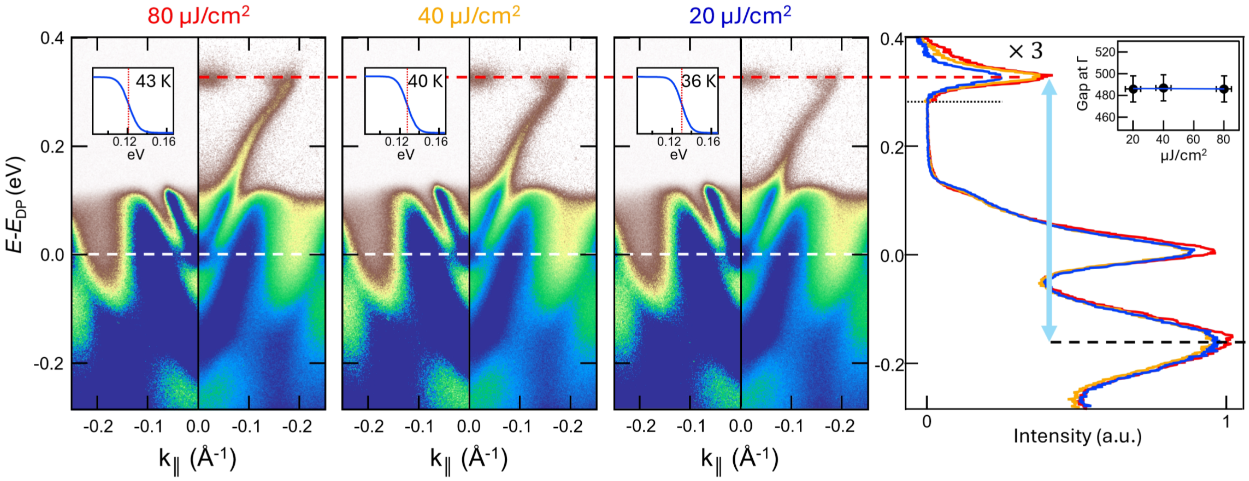

FIG. S6. **Fluence dependence of the energy gap at $\mathbf{\Gamma}$.** TR-ARPES intensity maps at negative delay (left) and time-integrated (right; as in Fig. 2) for different pump fluences. The inset show Fermi–Dirac fits used to extract the electronic temperature. EDCs in the right panel show that the Γ-point gap remains constant across all fluences (see inset).

Ultrafast charge dynamics in the scattering phase space

To better interpret the observed ultrafast dynamics, Fig. S7 shows the same experimental and simulated traces of Fig. 3d, but normalized in intensity. At 30 K, due to the minimal thermal phonon population, the Γ valley exhibits a sharp rise followed by a fast decay, while the Q-valley population reaches its maximum only when the Γ valley is empty, thus with a certain delay. This delayed dynamic is well reproduced by the simulations and originates from inefficient Γ→Q intervalley scattering at low temperature, which then develops into slow dynamics at Q. At 180 K, increased phonon absorption combined with band structure renormalization enhances Γ-valley population by enabling the Q→Γ backscattering channel. Despite its simplicity, the KMC model captures the main experimental trends, supporting its validity at elevated temperatures.

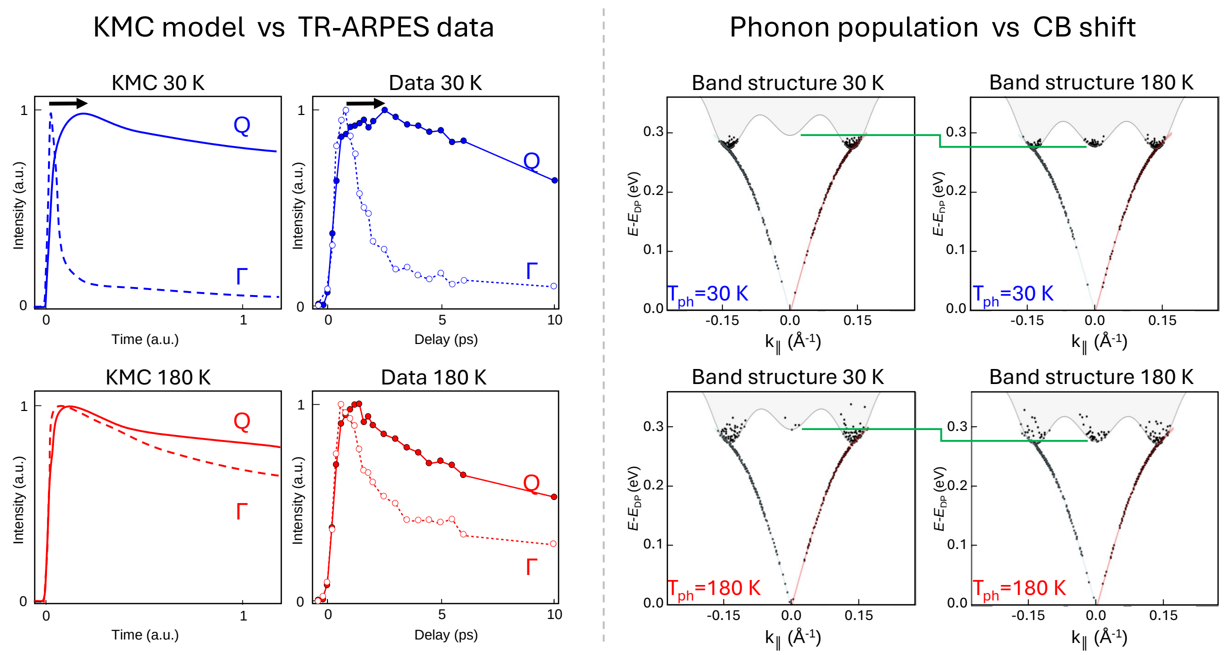

FIG. S7. **Ultrafast charge dynamics in the scattering phase space.** Contributions of the phonon population and band renormalization to the ultrafast dynamics. (Left) Comparison between KMC simulations and experimental traces at 30 K (blue) and 180 K (red), normalized in intensity to highlight the ultrafast dynamics at Γ (dashed) and Q (solid). (Right) Comparison between the calculated occupations for two phonon populations (T_{ph} = 30 K, blue; T_{ph} = 180 K, red) for the two experimentally-retrieved band structures (30 K and 180 K).

To decouple phonon population effects from band gap renormalization at high temperature, the right panel of Fig. S7 examines the impact of two thermal phonon distributions (T_{ph} = 30 K and T_{ph} = 180 K) on the carriers dynamics for the conduction band structures extracted experimentally at 30 K and 180 K. For the 30 K band structure, the Γ valley remains unoccupied even for a phonon population of T_{ph} = 180 K. In contrast, for the 180 K band structure (-16 meV shift at Γ), carriers populate the Γ valley already at T_{ph} = 30 K. These results demonstrate that the minor renormalization of the conduction band, which reflects changes in the electron–phonon scattering phase space, controls both the temperature evolution and the ultrafast dynamics of the intensity build-up. This is the reason why the Γ/Q intensity ratio follows the spectral overlap derived from the measured dispersions (Fig. 3a,b, main text). We emphasize that, in the simulations, the temperatures and time scales are effective parameters rather than physical quantities. Electron–phonon coupling, electron density, and grid density are phenomenologically tuned; accordingly, the intensity scale and the time axis are in arbitrary units.

[1] A. Longa, J.-M. Parent, B. K. Frimpong, D. Armanno, N. Gauthier, F. Légaré, F. Boschini, and G. Jargot, Optics Express **32**, 29549 (2024).

[2] N. Gauthier, J. A. Sobota, H. Pfau, A. Gauthier, H. Soifer, M. D. Bachmann, I. R. Fisher, Z. X. Shen, and P. S. Kirchmann, Review of Scientific Instruments **92**, 123907 (2021).

[3] S. Beaulieu, M. Schüler, J. Schusser, S. Dong, T. Pincelli, J. Maklar, A. Neef, F. Reinert, M. Wolf, L. Rettig, J. Minár, and R. Ernstorfer, npj Quantum Materials **6**, 93 (2021).

[4] C.-H. Min, H. Bentmann, J. N. Neu, P. Eck, S. Moser, T. Figgemeier, M. Ünzelmann, K. Kissner, P. Lutz, R. J. Koch, C. Jozwiak, A. Bostwick, E. Rotenberg, R. Thomale, G. Sangiovanni, T. Siegrist, D. Di Sante, and F. Reinert, Physical Review Letters **122**, 116402 (2019).

[5] Y. Cao, J. A. Waugh, X.-W. Zhang, J.-W. Luo, Q. Wang, T. J. Reber, S. K. Mo, Z. Xu, A. Yang, J. Schneeloch, G. D. Gu, M. Brahlek, N. Bansal, S. Oh, A. Zunger, and D. S. Dessau, Nature Physics **9**, 499–504 (2013).

[6] E. Papalazarou, L. Khalil, M. Caputo, L. Perfetti, N. Nilforoushan, H. Deng, Z. Chen, S. Zhao, A. Taleb-Ibrahimi, M. Konczykowski, A. Hruban, A. Wołoś, A. Materna, L. Krusin-Elbaum, and M. Marsi, Physical Review Materials **2**, 104202 (2018).

[7] R. Mori, K. Takasan, P. Ai, S. Ciocys, K. Kawaguchi, T. Kondo, T. Morimoto, and A. Lanzara, Proceedings of the National Academy of Sciences **122**, e2422667122 (2025).

[8] R. Mori, S. Ciocys, K. Takasan, P. Ai, K. Currier, T. Morimoto, J. E. Moore, and A. Lanzara, Nature **614**, 249 (2023).

[9] D. Singh, S. Nandi, Y. Fleger, S. Z. Cohen, and T. Lewi, Laser & Photonics Reviews **17**, 2200841 (2023).

[10] B. Monserrat and D. Vanderbilt, Physical Review Letters **117**, 226801 (2016).
